# Development and validation of a novel hepato-metabolic-renal score nomogram for predicting disease-free survival in head and neck squamous cell carcinoma

**DOI:** 10.3389/fonc.2026.1815660

**Published:** 2026-05-21

**Authors:** Lu Lu, Yue Chen, Shudong An, Nuoxuan Niu, Qian Chen, Xinyu Ban, Shuyan Liu, Shiyang Wang, Xiaorong Zhang, Yanqiang Wei, Lan Yao, Yanxia Bai

**Affiliations:** 1 Department of Anesthesiology and Surgery, The First Affiliated Hospital of Xi’an Jiaotong University, Xi’an, Shaanxi, China; 2 Center for Gut Microbiome Research, Med-X Institute Centre, The First Affiliated Hospital of Xi’an Jiaotong University, Xi’an, Shaanxi, China; 3 Xi’an Aeronautical Computing Technique Research Institute of AVIC, Xi’an, Shaanxi, China; 4 Department of Otorhinolaryngology Head and Neck Surgery, The First Affiliated Hospital of Xi’an Jiaotong University, Xi’an, Shaanxi, China

**Keywords:** disease-free survival, head and neck squamous cell carcinoma, nomogram, prognostic score, surgical oncology

## Abstract

**Background:**

Head and neck squamous cell carcinoma (HNSCC) exhibits marked heterogeneity in disease-free survival (DFS), highlighting the need for improved prognostic tools. This study aimed to develop and validate an integrative physiological score for surgical HNSCC patients.

**Methods:**

Using a single-center cohort of 321 surgical HNSCC patients randomly divided into training (n=225) and validation (n=96) cohorts, we developed the Hepato-Metabolic-Renal Score (HMRS) from 11 routine preoperative laboratory parameters via LASSO Cox regression. HMRS was integrated with clinicopathological factors into a prognostic nomogram. Performance was assessed by discrimination (time-dependent ROC), calibration, and clinical utility (decision curve analysis).

**Results:**

HMRS demonstrated significant prognostic value for DFS in both training (HR = 2.907, 95%CI:1.693–4.991, P = 0.001) and validation (HR = 2.356, 95% CI: 1.082-5.130, P = 0.031) cohorts. The nomogram showed excellent discrimination (1/3/5-year AUCs: 0.913/0.899/0.896 in training, 0.952/0.891/0.923 in validation), good calibration, and positive net benefit on decision curve analysis. Subgroup analyses confirmed consistent performance across clinical subsets without significant interaction effects after multiple comparison correction.

**Conclusions:**

The Hepato-Metabolic-Renal Score provides a novel, practical prognostic tool for surgical HNSCC, with its nomogram enabling personalized recurrence risk estimation. This integrative approach advances precision surgery strategies.

## Introduction

Head and neck squamous cell carcinoma (HNSCC) exhibits marked heterogeneity in disease-free survival (DFS) outcomes, with recurrence rates varying substantially even among patients with similar clinicopathological features (1, 2). This prognostic uncertainty poses significant challenges for treatment personalization and surveillance strategies, as current TNM-based staging systems fail to adequately capture the complex interplay between tumor biology and host physiological factors that determine recurrence risk (3, 4). The inability to accurately predict DFS trajectories represents a critical barrier to optimizing post-treatment surveillance intensity and implementing risk-adapted therapeutic approaches, highlighting an urgent need for improved prognostic tools in HNSCC management.

Recent advances in prognostic biomarker discovery have identified various molecular signatures (such as HPV status EGFR expression, and immune cell infiltration scores) (5–7), and imaging features (including radiomic signatures and PET metabolic parameters) (8, 9) associated with HNSCC outcomes. However, these approaches often require specialized assays or advanced imaging modalities that limit their widespread clinical adoption. Moreover, current HNSCC prediction models, like nomograms and risk scores, remain focused on tumor-intrinsic characteristics (e.g., tumor stage, nodal involvement, extracapsular extension) (10–12), with limited integration of systemic physiological parameters that may influence surgical outcomes. This gap between sophisticated biomarker research and practical clinical prediction represents a significant barrier to implementing truly personalized treatment approaches based on comprehensive assessment for surgical HNSCC patients.

For patients undergoing curative-intent surgery, systemic organ function—encompassing hepatic, metabolic, and renal homeostasis—represents a critical yet underutilized prognostic determinant. Impairments in these physiological systems, reflected by routine preoperative laboratory parameters, are associated with increased surgical morbidity and compromised recovery trajectories (13, 14). Furthermore, suboptimal physiological status may diminish the host’s capacity to mount effective antitumor responses during the critical perioperative period (15). We hypothesize that a composite assessment of these biomarkers could provide a novel measure of host physiological fitness with direct relevance to surgical oncological outcomes. To translate this physiological concept into a clinically applicable tool, we developed the Hepato-Metabolic-Renal Score (HMRS)—a novel composite measure derived from routine preoperative laboratory parameters. HMRS quantifies integrated organ function, providing an objective metric of host physiological fitness that complements existing tumor-centric prognostic factors.

To advance prognostic precision in surgical HNSCC, we designed a two-phase translational investigation. First, we aimed to develop and rigorously validate HMRS as a prognostic marker for DFS. Second, we sought to incorporate HMRS into a comprehensive nomogram alongside other independent prognostic factors. By bridging physiological insights with clinical prediction, this work contributes to the evolving paradigm of precision surgery—where treatment decisions are informed not only by tumor characteristics but also by comprehensive assessment of host physiological readiness and resilience.

## Patients and methods

### Study design and patient population

We conducted a retrospective cohort study of 321 patients with newly diagnosed, surgically treated head and neck squamous cell carcinoma (HNSCC) at the First Affiliated Hospital of Xi’an Jiaotong University between January 2014 and December 2020.

Inclusion criteria were: (1) Histologically confirmed HNSCC; (2) Curative-intent surgical resection with negative margins; (3) Availability of complete preoperative laboratory data within 2 weeks before surgery; (4) Minimum follow-up of 1 month.

Exclusion criteria were: (1) Distant metastasis at diagnosis; (2) Previous history of other malignancies; (3) Incomplete clinical or follow-up data; (4) Patients receiving neoadjuvant therapy.

Patients were randomly divided into training (n=225, 70%) and validation (n=96, 30%) cohorts using computer-generated random numbers, stratified by recurrence status to ensure balanced event distribution.

The study was conducted in accordance with the Declaration of Helsinki and approved by the ethics committee of First Affiliated Hospital of Xi’an Jiaotong University [No.2022-321]. All participants signed an informed consent.

### Data collection and variables

Clinical and pathological variables were extracted from electronic medical records: age, gender, smoking history, tumor site, TNM stage (AJCC 8th edition), histological differentiation, and treatment details. Comorbidity data, including hypertension, diabetes, chronic hepatitis, and chronic kidney disease, were also collected from medical records.

Laboratory parameters measured within 2 weeks before surgery included: Liver function: alanine aminotransferase (ALT), aspartate aminotransferase (AST), gamma-glutamyl transferase (γ-GGT), direct bilirubin (DBIL), indirect bilirubin (IDBIL), fibrinogen (FIB), albumin (Alb); Renal function: Cystatin C (Cys), creatinine (Cr); Metabolic markers: total cholesterol (TC); Inflammatory cells: neutrophil count (NEU), lymphocyte count (LYM), platelet count (PLT). Immune-inflammatory indices were calculated as follows: NLR = neutrophil count (×10^9^;/L)/lymphocyte count (×10^9^;/L); PLR = platelet count (×10^9^;/L)/lymphocyte count (×10^9^;/L); PNI= albumin (g/L) + 5 × lymphocyte count (×10^9^;/L); SII=platelet count (×10^9^;/L) × neutrophil count (×10^9^;/L)/lymphocyte count (×10^9^;/L); ALBI=(log_10_ bilirubin × 0.66) + (albumin × -0.085).

Outcome definition: Disease-free survival (DFS) was defined as the interval time (in months) from surgery to the first occurrence of any of the following events: local recurrence, regional recurrence, distant metastasis, or death from any cause. Patients without events were censored at the last follow-up date (December 2025).

### Hepato-metabolic-renal score development

Based on the rationale that integrated assessment of systemic organ function would enhance prognostic accuracy, we specifically focused on 11 routine laboratory parameters representing three physiological domains: hepatic function (FIB, Alb, ALT, AST, γ-GGT, DBIL, IDBIL, TBIL), metabolic status (TC) and renal function (Cys, Cr). The 11 candidate laboratory parameters were selected based on their biological relevance to hepatic, metabolic, and renal functions, routine clinical availability, and prior literature supporting their prognostic value in cancers (16, 17). Markers such as blood urea nitrogen and blood glucose were not included due to either lack of significant association with DFS in preliminary analysis or incomplete data availability.

LASSO Cox regression with 10-fold cross-validation was used to identify the most prognostically relevant variables from these candidate parameters. The selected variables and their coefficients were used to construct the HMRS. All variables were standardized (z-score normalization) before calculation. The HMRS was then categorized into high-risk and low-risk groups using the optimal cut-off value determined by survival-based cut-point analysis maximizing the log-rank statistic and maximizing the Youden index in receiver operating characteristic (ROC) analysis for 5-year DFS prediction.

Two sensitivity analyses were performed: (1) excluding variables with coefficients <0.01 to derive a simplified HMRS, and (2) replacing z-score standardization with a simple abnormal count based on clinical reference ranges. The performance of these alternative versions was compared with the original HMRS using time-dependent area under the curve (AUC).

### Nomogram development and validation

A nomogram was developed based on independent prognostic factors identified in multivariable Cox regression. The nomogram was evaluated in the training cohort and validated in the validation cohort. Performance was assessed in three dimensions: (1) Discriminative ability: Time-dependent ROC curves and area under AUC at 1, 3, and 5 years were calculated. (2) Calibration: Calibration curves were plotted to compare predicted and observed survival probabilities using 1000 bootstrap resamples. (3) Clinical utility: Decision curve analysis was performed to evaluate the net clinical benefit across different threshold probabilities.

To assess the robustness of the nomogram and address potential overfitting concerns, internal validation was performed using 1000 bootstrap resamples and 10-fold cross-validation on the entire dataset. The bootstrap validation yielded bias-corrected C-index and calibration slope. The optimism estimate was calculated as the difference between the apparent and bootstrap-corrected performance.

To further demonstrate the clinical utility of our nomogram, we compared its performance with the platelet-to-albumin ratio (PAR)-based nomogram reported by Tsai et al. (2024) in the validation cohort.

### Statistical analysis

Continuous variables are presented as mean ± standard deviation or median (interquartile range) based on distribution, and compared using Student’s t-test or Mann-Whitney U test. Categorical variables are presented as frequencies (percentages) and compared using Chi-square or Fisher’s exact test.

For Survival analysis, Kaplan-Meier curves were plotted and compared using the log-rank test. Hazard ratios (HR) with 95% confidence intervals (CI) were estimated using univariable and multivariable Cox proportional hazards models.

We examined the prognostic value of HMRS in predefined subgroups stratified by TNM stage (I-II vs III-IV), N stage (N0 vs N+), age (<60 vs ≥60 years), gender (female vs male), and smoking history (non-smoker vs smoker). Formal interaction tests were performed to assess effect modification. For subgroup interaction analyses, P-values were adjusted for multiple comparisons using the Benjamini-Hochberg false discovery rate (FDR) method. Corrected P-values <0.05 were considered statistically significant.

All statistical analyses were performed using R software version 4.2.2 (R Foundation for Statistical Computing, Vienna, Austria). Two-sided P values <0.05 were considered statistically significant.

## Results

### Patient characteristics

A total of 321 patients with surgically treated HNSCC were included in this study. The mean age was 61.32 years (± 9.29), and 92.9% of patients were male. The most common primary tumor type was glottic laryngocarcinoma (51.4%). According to AJCC 8th edition staging system, the distribution was as follows: stage 0/I in 130 patients (40.5%), stage II in 44 patients (13.7%), stage III in 76 patients (23.7%), and stage IV in 71 patients (22.1%). Overall, 174 patients (54.2%) had early-stage disease (stage I-II) and 147 patients (45.8%) had advanced-stage disease (stage III-IV). Regarding comorbidities, hypertension was present in 35 patients (10.9%) and diabetes in 19 patients (5.9%). Chronic hepatitis and chronic kidney disease were observed in only one patient each (0.3%). The median follow-up time was 63 months (range: 1–143 months), during which 143 patients (44.5%) experienced disease recurrence or death.

Patients were randomly divided into training (n=225) and validation (n=96) cohorts. As shown in **Table 1**, the two cohorts were well-balanced with no significant differences in baseline demographic, clinicopathological, laboratory characteristics, or comorbidities (all P > 0.05). The recurrence rates were 44.4% in the training cohort and 44.8% in the validation cohort (P = 0.954). Detailed patient characteristics are provided in **Table 1**.

**Table 1:** Baseline clinicopathological characteristics of the entire study cohort.

Characteristic	Total Patients (N=321)	Training cohort (N=225)	Validation cohort (N=96)	P-value
Gender				0.488
Male	296 (92.2%)	209 (92.9%)	87 (90.6%)	
Female	25 (7.8%)	16 (7.1%)	9 (9.4%)	
Age (year)	61.32±9.29	60.82±9.19	62.48±9.47	0.144
Smoking history				0.451
Non-smoker	105 (65.2%)	61 (27.1%)	30 (31.3%)	
Smoker	56 (34.8%)	164 (72.9%)	66 (68.8%)	
Comorbidities				
Hypertension	35 (10.9)	26 (11.6)	9 (9.4)	0.705
Diabetes	19 (5.9)	13 (5.8)	6 (6.3)	1
Tumor type				0.550
Laryngeal cancer (glottic)	165 (51.4%)	106 (47.1%)	52 (54.2%)	
Laryngeal cancer (supraglottic)	55 (17.1%)	43 (19.1%)	16 (16.7%)	
Laryngeal cancer (subglottic)	22 (6.9%)	13 (5.8%)	7 (7.3%)	
Other types	79 (24.6%)	63 (28.0%)	21 (21.9%)	
Tumor differentiation				0.193
Well differentiated	118 (36.8%)	79 (35.1%)	39 (40.6%)	
Moderately differentiated	140 (43.6%)	96 (42.7%)	44 (41.9%)	
Poorly differentiated	63 (19.6%)	50 (22.2%)	13 (13.5%)	
T stage				0.426
Tis/T1	137 (42.7%)	92 (40.9%)	45 (46.9%)	
T2	69 (21.5%)	47 (20.9%)	22 (22.9%)	
T3	83 (25.9%)	60 (26.7%)	23 (24.0%)	
T4	32 (10.0%)	26 (11.6%)	6 (6.3%)	
N stage				0.129
N0	224 (69.8%)	150 (66.7%)	74 (77.1%)	
N1	45 (14.0%)	33 (14.7%)	12 (12.5%)	
N2	52 (16.2%)	42 (18.7%)	10 (10.4%)	
M stage				
M0	315 (98.1%)	219 (97.3%)	96 (100.0%)	0.106
M1	6 (1.9%)	6 (2.7%)		
TNM stage (AJCC, 8th)				0.105
0/I	130 (40.5%)	88 (39.1%)	42 (38.9%)	
II	44 (13.7%)	30 (13.3%)	14 (14.6%)	
III	76 (23.7%)	49 (21.8%)	27 (28.1%)	
IV	71 (22.1%)	58 (25.8%)	13 (13.5%)	
LYM (10^9^/L)	1.650 (1.265-2.105)	1.650 (1.250-2.130)	1.676±0.574	0.700 *
NEU (10^9^/L)	3.780 (2.930-5.215)	3.720 (2.930-5.265)	3.935 (2.985-5.020)	0.645 *
PLT (10^9^/L)	186.0 (150.5-235.5)	189.0 (149.5-243.0)	178 (154.3-221.0)	0.333 *
FIB	3.110 (2.615-3.815)	3.180 (2.665-3.870)	3.05 (2.51-3.72)	0.340 *
Alb	39.90 (37.75-42.50)	39.90 (38.00-42.49)	39.90±4.12	0.823 *
ALT	18.59 (13.00-28.00)	19.00 (12.00-29.00)	18.00 (14.00-25.00)	0.724 *
AST	19.00 (15.75-23.50)	19.00 (15.00-24.00)	19.00 (16.00-23.00)	0.659 *
DBIL	3.10 (2.20-4.35)	3.10 (2.20-4.30)	3.15 (2.33-4.58)	0.683 *
IDBIL	7.60 (5.66-10.20)	7.50 (5.66-10.10)	7.75 (5.50-11.15)	0.696 *
TBIL	11.20 (8.20-14.40)	11.20 (8.25-14.35)	11.20 (8.13-14.88)	0.884 *
TC	4.25 (3.73-4.85)	4.29 (3.72-5.00)	4.17 (3.74-4.52)	0.107 *
γGGT	21.00 (15.00-34.00)	23.00 (16.00-35.00)	19.70 (13.00-28.00)	0.052 *
Cr	63.00 (56.25-72.00)	63.00 (56.25-71.25)	66.45±15.08	0.329 *
Cys	0.890 (0.780-1.010)	0.880 (0.767-1.000)	0.92±0.17	0.117 *
NLR	2.31 (1.58-3.34)	2.32 (1.53-3.29)	2.26 (1.67-3.38)	0.533 *
PLR	113.91 (85.78-156.64)	115.03 (85.95-155.80)	110.01 (85.41-160.58)	0.621 *
PNI	48.35 (44.65-52.30)	48.55 (44.81-52.35)	48.36±5.75	0.677 *
SII	343.15 (189.92-567.77)	323.68 (179.09-556.43)	387.75 (237.36-615.49)	0.096 *
ALBI	-4.07 (-4.32- -3.86)	-4.07 (-4.32- -3.88)	-4.09±0.40	0.818 *
HMRS (as continuous)	1.219 (0.699-1.609)	1.199 (0.695-1.654)	1.225 (0.702-1.532)	0.714 *
HMRS (dichotomized)				0.548 *
Low (<1.167)	135 (42.1%)	109 (48.4%)	43 (44.8%)	
High (≥1.167)	186 (57.9%)	116 (51.6%)	53 (55.2%)	

*Wilcoxon rank sum test, others are Chi-square test. Data are represented as mean (SD), median (interquartile range) or number (%).

### Development of the hepato-metabolic-renal score

**Figure 1:**
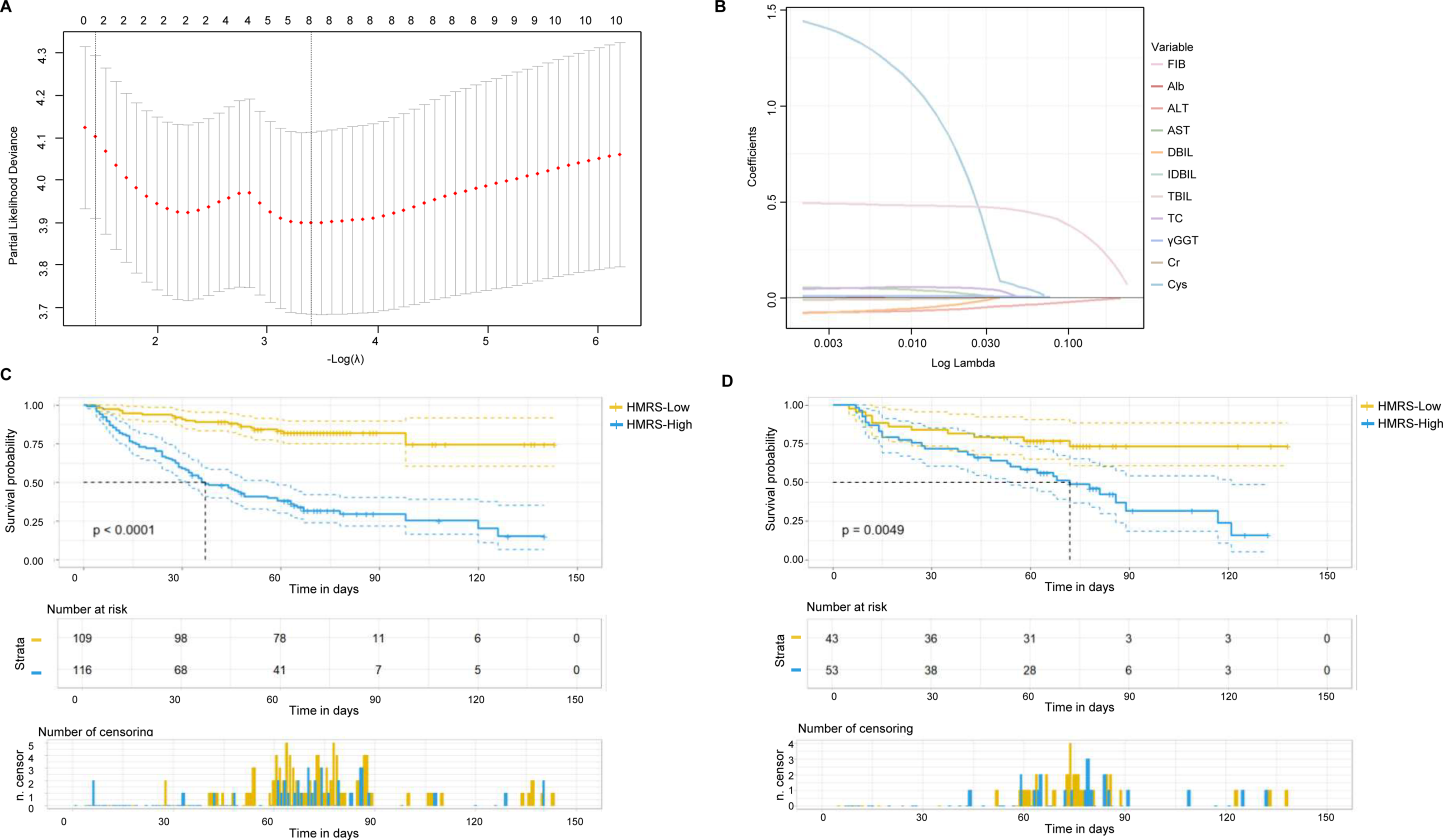
Development and validation of the hepato-metabolic-renal score (HMRS). **(A)** LASSO coefficient profiles of 11 candidate laboratory parameters. Each curve represents the coefficient path of one variable as the penalty parameter (λ) increases. **(B)** 10-fold cross-validation for tuning parameter (λ) selection in the LASSO Cox regression. **(C, D)** Kaplan-Meier curves for disease-free survival **(DFS)** stratified by the optimal HMRS cut-off value (1.167) in the training **(C)** and validation **(D)** cohorts. Log-rank P-values are displayed.

Using LASSO Cox regression with 10-fold cross-validation, eight variables were selected from the 11 candidate laboratory parameters: FIB, Cys, Cr, TC, DBIL, ALT, AST, and γ-GGT (**Figures 1A, B**). The HMRS was calculated using the following formula:

HMRS = 0.473379968 × FIBz + 0.532873248 × Cysz - 0.004039092 × Crz + 0.047986397 × TCz - 0.02376649 × DBILz - 0.055466398 × ALTz + 0.015301716 × ASTz + 0.005657624 × γ-GGTz, where subscript “z” indicates standardized values (z-scores).

The optimal cut-off value was 1.167, determined by maximizing the Youden index, corresponding to a specificity of 0.720 and sensitivity of 0.800. Receiver operating characteristic (ROC) analysis demonstrated excellent discriminative ability of HMRS for 5-year DFS prediction, with an area under the curve (AUC) of 0.835 (95% CI: 0.784-0.885) (**Supplementary Figure 1**).

### Survival outcomes stratified by HMRS

Stratification by the optimal HMRS cut-off value (1.167) effectively discriminated patients with distinct DFS outcomes. In the training cohort, patients with high HMRS (≥1.167) had significantly worse DFS compared to those with low HMRS (<1.167) (5-year DFS: 31.0% vs 81.7%, log-rank P < 0.0001; **Figure 1C**). This prognostic stratification was successfully validated in the internal validation cohort (5-year DFS: 39.6% vs 74.4%, log-rank P = 0.0049; **Figure 1D**).

### Comparison with traditional inflammatory indices

**Figure 2:**
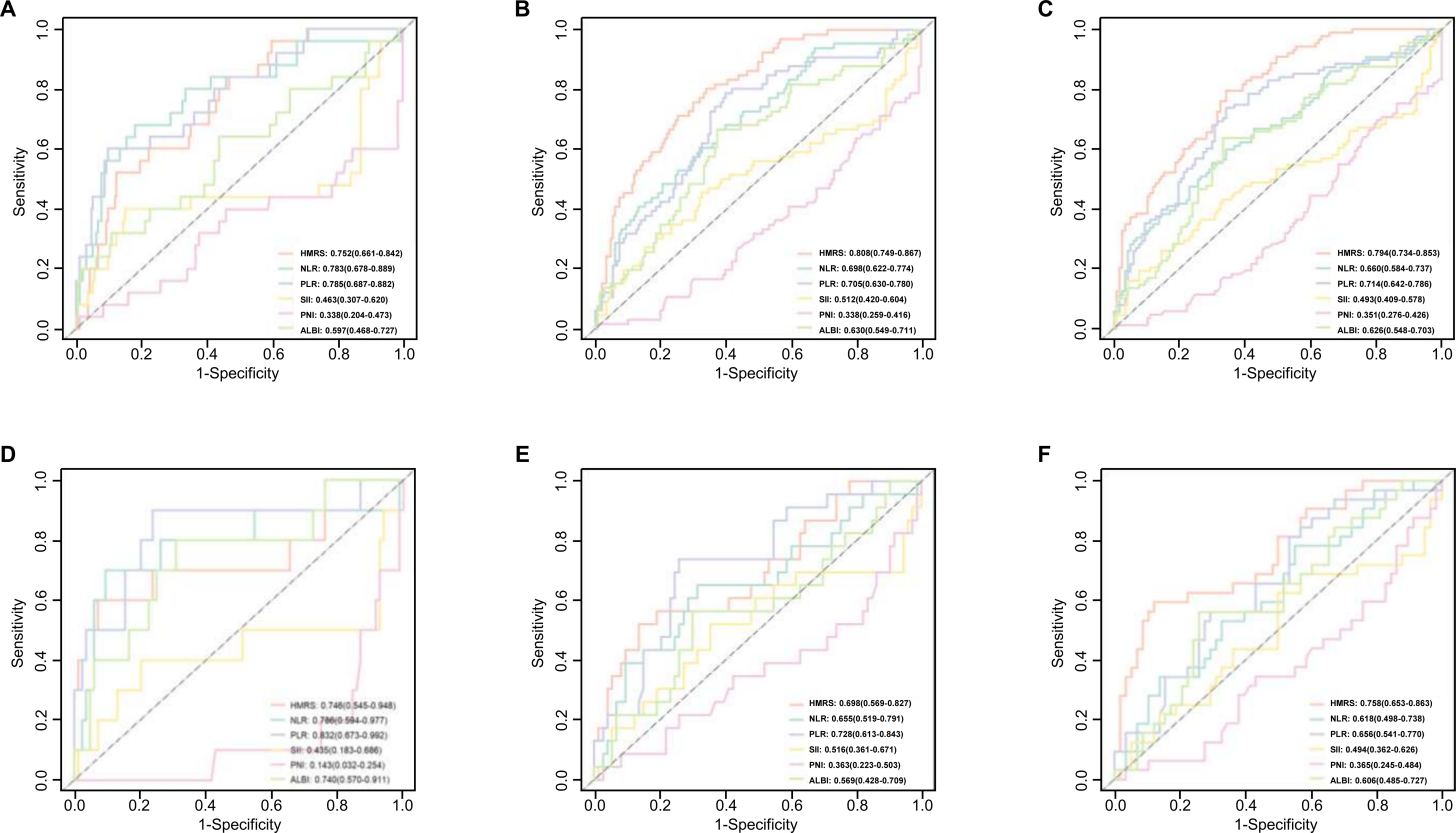
Comparison of prognostic accuracy between the HMRS and traditional inflammatory indices. **(A-C)** Time-dependent receiver operating characteristic (ROC) curves for predicting 1−, 3−, and 5−year DFS in the training cohort. **(D-F)** Corresponding ROC curves in the validation cohort. In all panels, the HMRS (continuous score) is compared with traditional indices: neutrophil−to−lymphocyte ratio (NLR), platelet−to−lymphocyte ratio (PLR), systemic immune−inflammation index (SII), prognostic nutritional index (PNI), and albumin−bilirubin grade (ALBI). The area under the curve (AUC) for each index is displayed.

The prognostic accuracy of the continuous HMRS score was first evaluated against traditional inflammatory indices using ROC analysis, which allows for direct comparison without the influence of a predefined cutoff. In the training cohort, the HMRS showed strong long-term predictive value, achieving the highest AUCs at 3 years (0.808) (**Figure 2B**) and 5 years (0.794) (**Figure 2C**), whereas at 1 year its AUC (0.752) was slightly lower than those of NLR (0.783) and PLR (0.785) (**Figure 2A**). In the validation cohort, the HMRS performed best at 5 years (AUC: 0.758) (**Figure 2F**), while at earlier timepoints it was outperformed by certain traditional indices—specifically PNI (1 year: 0.857), PLR (1 year: 0.832; 3 years: 0.728) (**Figures 2D, E**)., and NLR (1 year: 0.786) (**Figure 2D**).

### Sensitivity analyses

We performed two sensitivity analyses in both cohorts to assess model robustness (**Supplementary Table 1**; **Supplementary Figure 2**). First, excluding variables with coefficients <0.01 (Cr and γ-GGT) yielded a simplified 6-variable HMRS that maintained significant risk stratification in both cohorts (training: log-rank P < 0.0001; validation: log-rank P = 0.00018; **Supplementary Figures 2A, C**), albeit with slightly lower time-dependent AUCs compared to the original HMRS. Second, replacing z-score standardization with a simple abnormal count (0–8) resulted in substantially poorer discriminative ability and failed to achieve significant stratification in the validation cohort (log-rank P = 0.64; **Supplementary Figures 2B, D**), confirming that simple dichotomization loses important prognostic information and justifying the use of continuous z-score standardization.

### Subgroup analyses

We evaluated both the distribution and prognostic consistency of HMRS across clinically relevant subgroups.

First, the distribution of continuous HMRS scores was compared. In both cohorts, significantly higher HMRS values were observed in patients with advanced disease characteristics, including advanced TNM stage (III-IV) (**Supplementary Figures 3A, F**) and lymph node involvement (N+ stage) (**Supplementary Figures 3B, G**). In the training cohort, no significant differences in HMRS distribution were found across subgroups defined by gender, age, or smoking history (**Supplementary Figures 3C–E**). In the validation cohort, HMRS values did not differ significantly by gender or smoking status (**Supplementary Figures 3H, J**), but were significantly higher in younger patients (<60 years) compared to older patients (≥60 years) (**Supplementary Figure 3I**).

Second, Kaplan-Meier analysis assessed the consistency of dichotomized HMRS (cut-off: 1.167) across subgroups. In the training cohort, HMRS-High was significantly associated with worse DFS across all subgroups examined (all log-rank P < 0.01; **Supplementary Figures 4A–E**). In the validation cohort, this association remained significant in most subgroups, though statistical significance was not reached in the advanced TNM stage, N+ stage, younger age (<60 years), and smoker subgroups (**Supplementary Figures 4F–J**).

Formal interaction analysis revealed no statistically significant effect modification between HMRS and any subgroup variable after correction for multiple comparisons in either cohort (all FDR-corrected P for interaction > 0.05; **Supplementary Figure 5**), supporting its broad clinical applicability in diverse HNSCC populations.

### Independent prognostic factors identification

In the training cohort, univariable Cox regression analysis identified multiple factors significantly associated with DFS (P < 0.05), including HMRS, clinicopathological features (tumor type, differentiation, T/N/M/TNM stage), and serum/inflammatory indices (**Table 2**). Hypertension and diabetes were not associated with DFS (both P > 0.05). Similar results were observed in the validation cohort (**Table 3**).

**Table 2:** Univariate and multivariate analyses of DFS in training cohort according to clinicopathological factors.

Characteristic	Univariate analysis		Multivariate analysis	
	HR (95% CI)	P-value	HR (95% CI)	P-value
Gender				
Female vs. Male	1.111 (0.515-2.400)	0.788		
Age (year)	1.022 (0.999-1.045)	0.061		
Smoking history				
Non-smoker vs. Smoker	1.247 (0.792-1.963)	0.340		
Hypertension				
No vs. Yes	0.770 (0.388-1.528)	0.454		
Diabetes				
No vs. Yes	1.312 (0.637-2.705)	0.462		
Tumor type				
Laryngeal cancer (glottic)	Ref	<0.001		
Laryngeal cancer (supraglottic)	3.547 (2.009-6.263)	<0.001		
Laryngeal cancer (subglottic)	5.508 (2.689-11.283)	<0.001		
Other types	4.857 (2.915-8.093)	<0.001		
Tumor differentiation				
Well differentiated	Ref	<0.001	Ref	<0.001
Moderately differentiated	2.250 (1.326-3.816)	0.003	1.605 (0.927-2.780)	0.091
Poorly differentiated	5.927 (3.431-10.237)	<0.001	3.734 (2.052-6.793)	<0.001
T stage				
Tis/T1	Ref	<0.001		
T2	7.336 (3.297-16.325)	<0.001		
T3	17.674 (8.514-36.688)	<0.001		
T4	21.134 (9.448-47.273)	<0.001		
N stage				
N0	Ref	<0.001		
N1	4.794 (2.837-8.103)	<0.001		
N2	6.605 (4.123-10.582)	<0.001		
M stage				
M0 vs. M1	2.754 (1.117-6.788)	0.028		
TNM stage (AJCC, 8th)				
0/I	Ref	<0.001	Ref	<0.001
II	4.925 (1.904-12.739)	0.001	2.805 (1.052-7.478)	0.039
III	16.352 (7.411-36.079)	<0.001	9.759 (4.258-22.367)	<0.001
IV	22.567 (10.319-49.349)	<0.001	9.952 (4.293-23.072)	<0.001
LYM	0.585 (0.427-0.802)	0.001		
NEU	1.074 (1.025-1.125)	0.003		
PLT	1.006 (1.003-1.009)	<0.001		
FIB	1.803 (1.506-2.158)	<0.001		
Alb	0.932 (0.886-0.979)	0.005		
ALT	0.946 (0.926-0.967)	<0.001		
AST	0.964 (0.933-0.996)	0.026		
DBIL	0.923 (0.830-1.027)	0.140		
IDBIL	0.969 (0.920-1.021)	0.240		
TBIL	0.967 (0.928-1.007)	0.103		
TC	1.095 (0.899-1.332)	0.368		
γGGT	1.004 (0.999-1.010)	0.114		
Cr	1.000 (0.997-1.004)	0.801		
Cys	1.294 (0.982-1.705)	0.067		
NLR	1.074 (1.044-1.104)	<0.001		
PLR	1.004 (1.003-1.005)	<0.001	1.002 (1.001-1.004)	0.001
PNI	0.927 (0.894-0.961)	<0.001		
SII	1.000 (1.000-1.000)	0.958		
ALBI	2.128 (1.288-3.517)	0.003		
HMRS (as continuous)	3.321 (2.553-4.322)	<0.001		
HMRS (dichotomized)				
Low (<1.167) vs. High (≥1.167)	5.531 (3.381-9.048)	<0.001	2.907 (1.693-4.991)	0.001

**Table 3:** Univariate and multivariate analyses of DFS in validation cohort according to clinicopathological factors.

Characteristic	Univariate analysis		Multivariate analysis	
	HR (95% CI)	P-value	HR (95% CI)	P-value
Gender				
Female vs. Male	1.572 (0.612-4.038)	0.347		
Age (year)	1.034 (0.998-1.072)	0.064		
Smoking history				
Non-smoker vs. Smoker	1.264 (0.657-2.433)	0.482		
Hypertension				
No vs. Yes	0.452 (0.109-1.872)	0.274		
Diabetes				
No vs. Yes	1.634 (0.501-5.329)	0.415		
Tumor type				
Laryngeal cancer (glottic)	Ref	0.001		
Laryngeal cancer (supraglottic)	2.340 (0.985-5.560)	0.054		
Laryngeal cancer (subglottic)	2.125 (0.702-6.434)	0.182		
Other types	4.477 (2.193-9.143)	<0.001		
Tumor differentiation				
Well differentiated	Ref	0.001	Ref	0.050
Moderately differentiated	4.398 (1.996-9.694)	<0.001	3.054 (1.186-7.864)	0.021
Poorly differentiated	4.345 (1.664-11.342)	0.003	1.531 (0.488-4.805)	0.466
T stage				
Tis/T1	Ref	<0.001		
T2	5.813 (2.074-16.295)	0.001		
T3	11.943 (4.683-30.461)	<0.001		
T4	24.781 (7.542-81.428)	<0.001		
N stage				
N0	Ref	<0.001		
N1	3.940 (1.739-8.927)	0.001		
N2	8.376 (3.768-18.620)	<0.001		
TNM stage (AJCC, 8th)				
0/I	Ref	<0.001	Ref	0.004
II	5.860 (1.533-22.394)	0.010	6.189 (1.423-26.926)	0.015
III	14.286 (4.812-42.412)	<0.001	7.601 (2.244-25.747)	<0.001
IV	39.419 (12.155-127.841)	<0.001	21.275 (3.896-116.176)	<0.001
LYM	0.572 (0.318-1.030)	0.053		
NEU	1.110 (0.946-1.302)	0.200		
PLT	1.004 (1.001-1.008)	0.023		
FIB	2.622 (1.791-3.839)	<0.001		
Alb	0.931 (0.862-1.007)	0.073		
ALT	0.948 (0.912-0.985)	0.006		
AST	0.944 (0.895-0.994)	0.030		
DBIL	0.942 (0.759-1.167)	0.583		
IDBIL	0.937 (0.867-1.013)	0.101		
TBIL	0.949 (0.893-1.009)	0.092		
TC	0.864 (0.587-1.273)	0.460		
γGGT	1.000 (0.992-1.009)	0.936		
Cr	0.991 (0.971-1.010)	0.352		
Cys	4.304 (0.685-27.041)	0.120		
NLR	1.079 (0.988-1.179)	0.092		
PLR	1.009 (1.005-1.013)	<0.001	1.007 (1.002-1.013)	0.013
PNI	0.933 (0.880-0.990)	0.022		
SII	1.000 (0.999-1.001)	0.873		
ALBI	2.146 (0.977-4.713)	0.057		
HMRS (as continuous)	6.663 (3.519-12.615)	<0.001		
HMRS (dichotomized)				
Low (<1.167) vs. High (≥1.167)	2.577 (1.297-5.119)	0.007	2.356 (1.082-5.130)	0.031

Of note, HMRS showed strong prognostic value both as a continuous variable (HR = 3.321, 95% CI: 2.553-4.322, P < 0.001) and when dichotomized at the optimal cut-off of 1.167 (HR = 5.531, 95% CI: 3.381-9.048, P < 0.001). For subsequent multivariable analysis. we selected the dichotomized form of HMRS due to its enhanced clinical applicability.

The multivariable model confirmed dichotomized HMRS, tumor differentiation, TNM stage, and PLR as independent predictors (**Table 2**). Specifically, high HMRS (HR = 2.907, 95%CI:1.693–4.991, P = 0.001), poor differentiation (vs. well differentiated: HR = 3.734, 95%CI:2.052-6.793, P<0.001), advanced TNM stage (e.g., Stage IV vs. 0/I: HR = 9.952, 95%CI:4.293-23.072, P<0.001), and elevated PLR (HR = 1.002, 95%CI:1.001–1.004, P = 0.001) were associated with worse DFS.

These findings were further validated in the internal validation cohort. In the multivariable model, dichotomized HMRS (HR = 2.356, 95% CI: 1.082-5.130, P = 0.031), tumor differentiation, TNM stage, and PLR remained significant independent prognostic factors for worse DFS.

The consistent identification of dichotomized HMRS as an independent predictor in both cohorts underscores its robust prognostic value in HNSCC.

### Nomogram construction and performance evaluation

**Figure 3:**
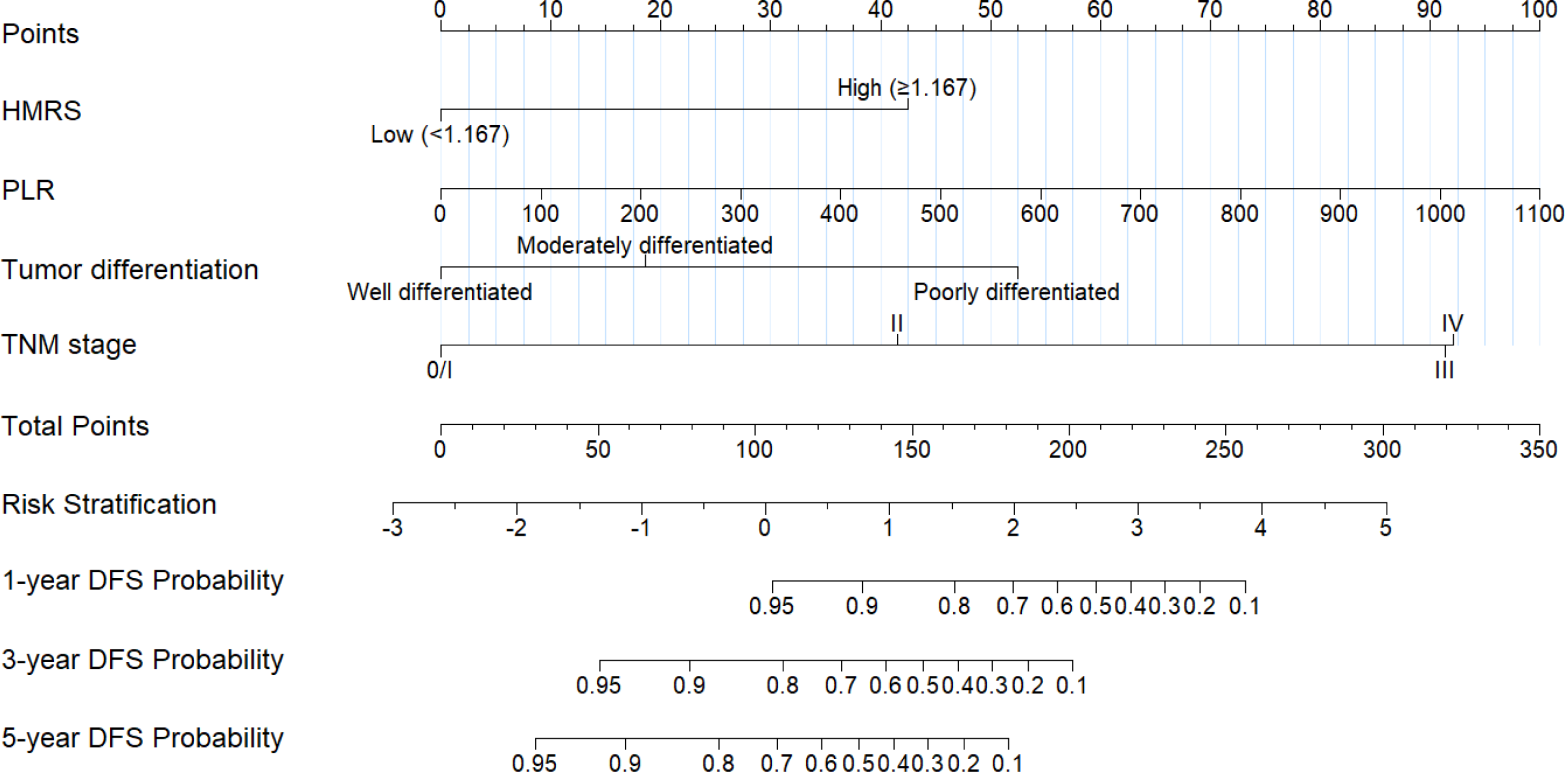
Prognostic nomogram for predicting DFS in HNSCC. The nomogram was constructed based on four independent predictors identified by multivariable Cox regression: dichotomized HMRS, tumor differentiation, TNM stage, and PLR. To obtain an individual patient’s probability of 1−, 3−, or 5−year DFS, first locate the patient’s value on each variable axis, draw a vertical line upward to the “Points” axis to obtain the corresponding score, sum all points, and finally draw a vertical line downward from the “Total Points” axis to the survival probability axes at the bottom.

Based on multivariable Cox regression results, a prognostic nomogram was constructed incorporating the four identified independent predictors: HMRS (dichotomized), tumor differentiation, TNM stage and PLR (**Figure 3**). The nomogram assigns specific points to each variable level, with total points corresponding to predicted 1-, 3-, and 5-year DFS probabilities. Based on the total nomogram points, patients were stratified into distinct risk categories for DFS, with scores ranging from -3 to 5.

### Nomogram performance

The nomogram demonstrated excellent discriminative ability with concordance index (C-index) values of 0.841(95% CI: 0.808-0.873) in the training cohort and 0.862 (95% CI: 0.813-0.909) in the validation cohort.

**Figure 4:**
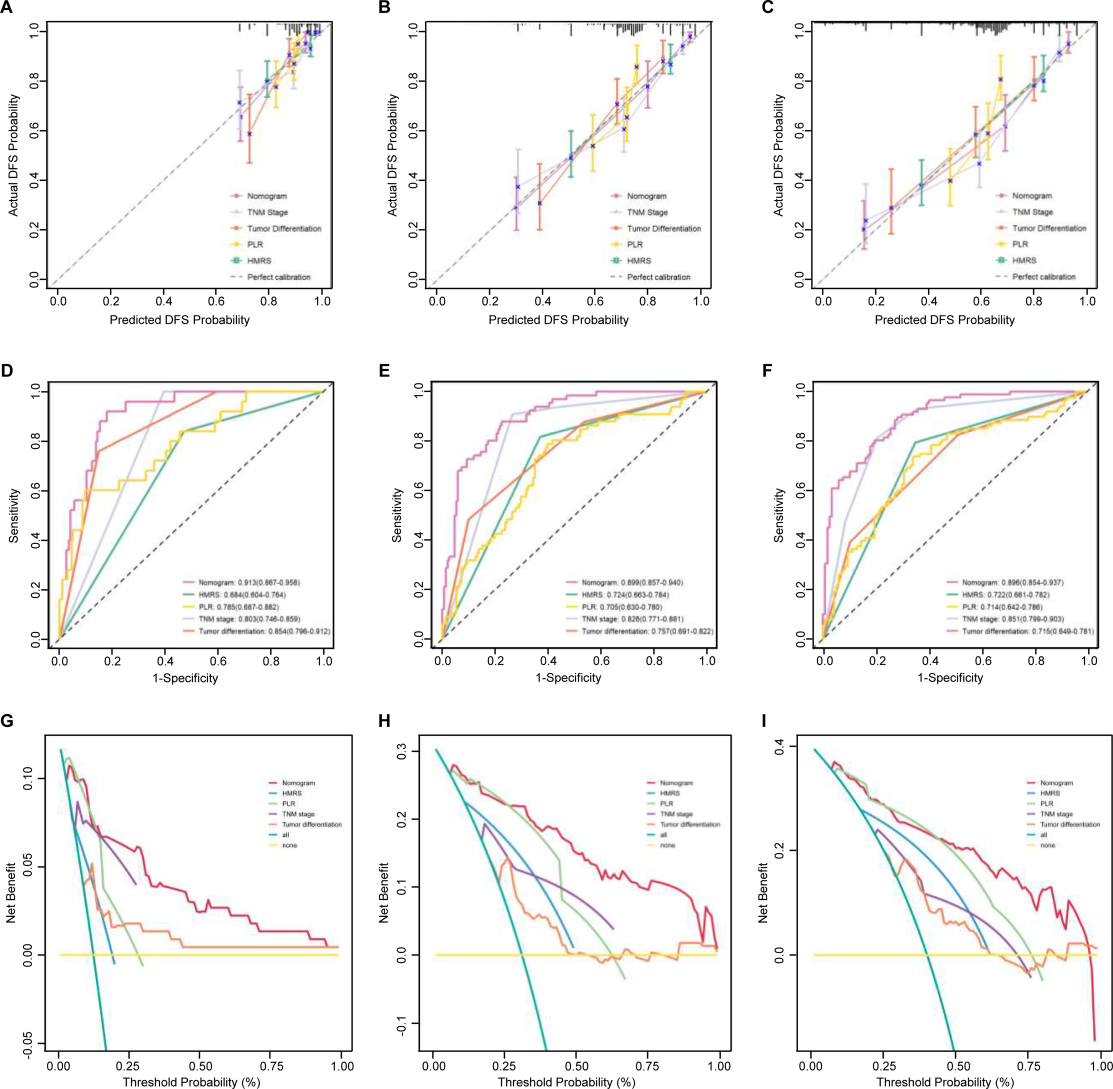
Performance and clinical utility of the nomogram in comparison to individual predictors in the training cohort. **(A-C)** Calibration curves assessing agreement between predicted and observed survival probabilities at **(A)** 1-year, **(B)** 3-year, and **(C)** 5-year follow-up. **(D-F)** Time-dependent ROC curves evaluating discrimination ability at **(D)** 1-year, **(E)** 3-year, and **(F)** 5-year. **(G-I)** Decision curve analysis (DCA) quantifying clinical net benefit at **(G)** 1-year, **(H)** 3-year, and **(I)** 5-year. The nomogram’s performance is compared against models containing only one single variable: TNM stage, tumor differentiation, HMRS, or PLR.

Calibration curves showed good agreement between nomogram-predicted and observed survival probabilities at 1, 3 and 5 years in both the training (**Figures 4A–C**) and validation (**Supplementary Figures 6A–C**) cohort, with predictions closely aligned along the ideal 45-degree line. This visual assessment, combined with the high C−index values, confirms that the nomogram is well-calibrated without evident overfitting compared to models containing only individual variables (dichotomized HMRS, TNM stage, tumor differentiation, or PLR alone).

In time−dependent ROC analysis, the nomogram achieved high AUC values for DFS prediction: 0.913, 0.899, and 0.896 at 1, 3, and 5 years, respectively, in the training cohort (**Figures 4D–F**); and 0.952, 0.891, and 0.923 in the validation cohort (**Supplementary Figures 6D–F**). These values were consistently superior to those of the individual constituent variables.

Finally, decision curve analysis (DCA) confirmed the superior clinical net benefit of the nomogram across a wide range of threshold probabilities for 1−, 3−, and 5−year DFS predictions. The nomogram outperformed both the strategy of using any single variable and the traditional “treat−all” or “treat−none” approaches in both the training (**Figures 4G–I**) and validation (**Supplementary Figures 6G–I**) cohorts.

### Assessment of model robustness

To assess the robustness of the nomogram and address potential overfitting concerns, we performed internal validation using 1000 bootstrap resamples and 10-fold cross-validation on the entire dataset (N = 321). The bootstrap validation yielded a bias-corrected C-index of 0.849, a calibration slope of 0.904, and a minimal optimism estimate of 0.010, confirming no evidence of overfitting. Ten-fold cross-validation further supported these findings, with a C-index of 0.858 and a calibration slope of 1.117 (**Supplementary Table 2**).

### Comparison with existing prognostic models

**Figure 5:**
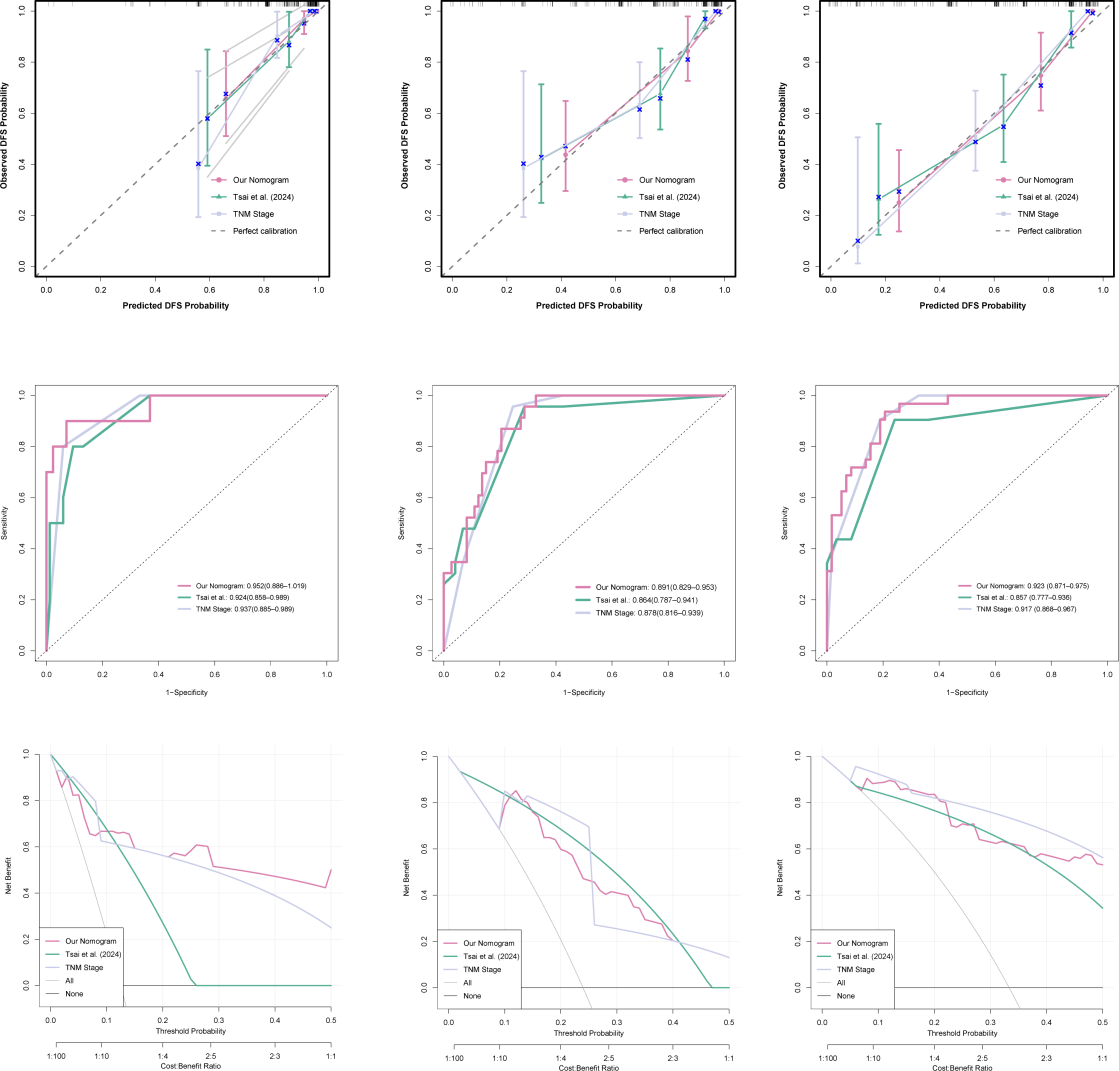
Comparison of the proposed nomogram with existing prognostic models in the validation cohort. **(A–C)** ROC curves with AUC values at 1, 3, and 5 years. **(D–F)** Calibration curves at 1, 3, and 5 years; the dashed line indicates ideal calibration. **(G–I)** Decision curve analysis at 1, 3, and 5 years. Our nomogram outperformed the platelet-to-albumin ratio (PAR)-based nomogram (Tsai et al., 2024) and the AJCC 8th TNM staging system across all evaluation metrics.

We further compared our nomogram with the PAR-based nomogram reported by Tsai et al. (2024) in the validation cohort. Our nomogram demonstrated superior discriminative ability at all time points, achieving time-dependent AUCs of 0.952 (1-year), 0.891 (3-year), and 0.923 (5-year), compared to 0.924, 0.864, and 0.857 for the Tsai et al. model (**Figure 5**).

## Discussion

In this study, we developed and validated the Hepato-Metabolic-Renal Score (HMRS) and its integration into a prognostic nomogram. HMRS demonstrated significant prognostic value for DFS in both cohorts (training cohort: HR = 2.907, 95%CI:1.693–4.991, P = 0.001; validation cohort: HR = 2.356, 95% CI: 1.082-5.130, P = 0.031), and the nomogram showed excellent performance across discrimination (training cohort: 1-year AUC = 0.913, 3-year AUC = 0.899, 5-year AUC = 0.896; validation cohort: 1-year AUC = 0.952, 3-year AUC = 0.891, 5-year AUC = 0.923), calibration, and clinical utility metrics. Subgroup analyses further confirmed HMRS’s consistent prognostic performance across various clinical subsets without significant interaction effects after multiple comparison adjustment.

HMRS represents more than a prognostic score—it embodies a paradigm shift toward integrative physiological assessment in surgical oncology (18, 19). Traditional prognostic models conceptualize cancer outcomes primarily through tumor-intrinsic properties, while HMRS introduces the critical dimension of host physiological fitness. This integration recognizes that surgical outcomes emerge from dynamic interactions between tumor biology and host resilience. The specific inclusion of hepatic, metabolic, and renal parameters reflects their synergistic roles in perioperative physiology: hepatic function regulates acute-phase responses (20); metabolic status influences recovery capacity (21); renal clearance modulates inflammatory homeostasis (22). HMRS translates this integrative concept into a clinically applicable metric, providing a host-focused prognostic dimension that complements conventional tumor-centric staging. Future research should investigate the specific biological pathways through which integrated physiological function influences recurrence risk, including perioperative immune-metabolic profiling and dynamic assessment of physiological recovery trajectories.

The biological rationale for including specific biomarkers such as cystatin C and fibrinogen is supported by their emerging roles in tumor progression. Cystatin C, a cysteine protease inhibitor, regulates cathepsin activity, which is crucial for extracellular matrix remodeling and tumor invasion (23). Elevated cystatin C levels have been associated with lymph node metastasis and poor prognosis in various cancers (24). Moreover, recent evidence indicates that cystatin C contributes to an immunosuppressive tumor microenvironment by recruiting Trem2-positive macrophages and modulating immune cell polarization (23, 25). Fibrinogen, an acute-phase protein frequently elevated in cancer patients, activates the IL-6/STAT3 signaling pathway, which drives epithelial-mesenchymal transition and promotes tumor cell migration and invasion (26). Furthermore, fibrinogen facilitates the formation of platelet-tumor cell aggregates, which physically shield tumor cells from natural killer cell-mediated immune surveillance (27). In head and neck squamous cell carcinoma specifically, elevated preoperative fibrinogen levels are independently associated with advanced TNM stage, lymph node metastasis, and poor disease-free survival (28). These mechanisms justify the inclusion of cystatin C and fibrinogen in the HMRS and provide a biological foundation for the score’s prognostic value.

Current prognostic models for HNSCC predominantly focus on tumor-intrinsic characteristics, with limited incorporation of host physiological factors (29, 30). Compared to molecular signatures requiring specialized assays or advanced imaging features necessitating complex analyses (31, 32), HMRS offers distinct practical advantages: it utilizes data already available in electronic health records, incurs no additional costs, and provides immediately actionable results. The absence of significant interaction effects between HMRS and all examined subgroups (including gender, age, smoking history, N stage and TNM stage; all FDR-corrected P>0.05 in both training and validation cohorts) further supports its general applicability across diverse patient populations. This consistency enhances the model’s potential for broad clinical implementation, as it does not appear to require subgroup-specific calibration.

The clinical implementation of HMRS and its associated nomogram could follow a straightforward pathway: automated calculation from existing laboratory data, integration with clinicopathological variables, and generation of individualized risk estimates to inform multidisciplinary discussions (33). For high-risk patients identified by HMRS, considerations might include intensified surveillance protocols (34), enrollment in adjuvant therapy trials, or preoperative physiological optimization (35). Conversely, low-risk patients might be candidates for reduced surveillance intensity (36) or consideration of treatment de-escalation strategies (37). The clinical utility of such risk-based stratification has been demonstrated in other diseases, including IgA nephropathy where polygenic risk scores enabled improved patient stratification and prognosis prediction (38). The nomogram’s calibration accuracy across the risk spectrum enhances its utility for personalized patient counseling regarding recurrence probabilities (39).

This study has several limitations warrant consideration. Methodologically, this study is limited by its retrospective, single-center design. Although we performed rigorous internal validation using bootstrap resampling and cross-validation, the lack of external validation from an independent multi-institutional cohort is a notable limitation. Therefore, our findings require prospective validation in diverse populations before clinical implementation, and claims regarding the model’s generalizability should be interpreted with caution. Clinically, the exclusive surgical focus may limit applicability to other treatment settings. Besides, data on adjuvant therapy (radiotherapy or chemoradiotherapy) were not available in our electronic medical records. Although all patients underwent curative-intent surgery, the absence of adjuvant treatment information precludes adjustment for this potential confounder. However, the primary aim of this study was to assess host physiological factors rather than treatment effects, and future prospective studies should collect these variables to further validate our findings. Biologically, while HMRS demonstrates prognostic value, underlying mechanisms need further exploration. Practically, inter-institutional laboratory variability may affect broader implementation. Temporally, optimal assessment timing remains undetermined. These limitations highlight directions for future research while not diminishing the current findings’ validity.

## Conclusions

In conclusion, the Hepato-Metabolic-Renal Score represents a practical prognostic tool that integrates routine physiological parameters for surgical HNSCC risk stratification. Its incorporation into a nomogram facilitates personalized recurrence prediction. Prospective multicenter validation is warranted to confirm its generalizability.

## Data Availability

The raw data supporting the conclusions of this article will be made available by the authors, without undue reservation.
