# Supplementary figures and images for: Development and validation of a novel hepato-metabolic-renal score nomogram for predicting disease-free survival in head and neck squamous cell carcinoma
Source: Front Oncol. 2026 May 21;16:1815660. doi: 10.3389/fonc.2026.1815660 (PMC13233239; doi:10.3389/fonc.2026.1815660)

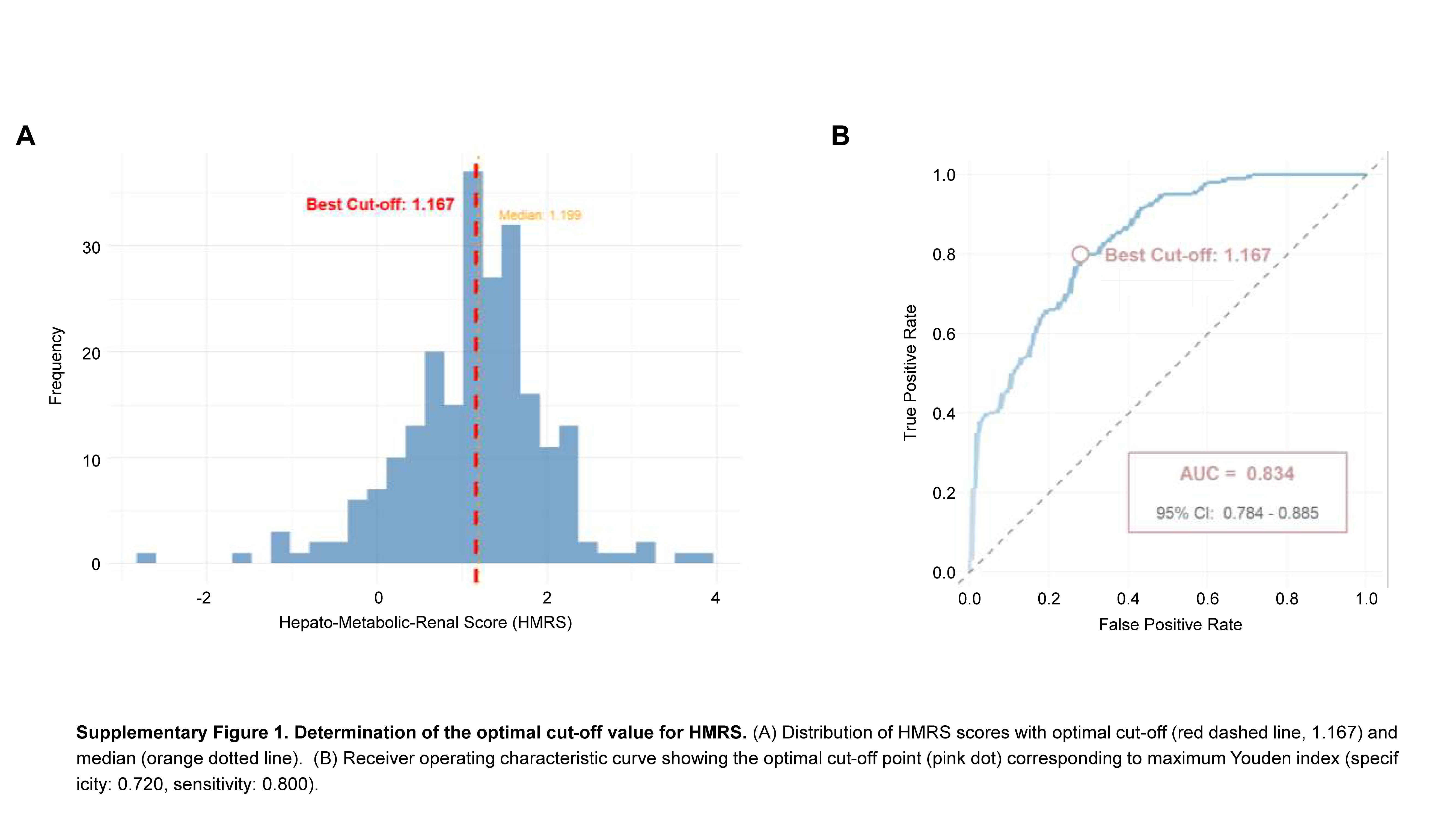

Supplement: Supplementary file 1 [file Image1.tif]

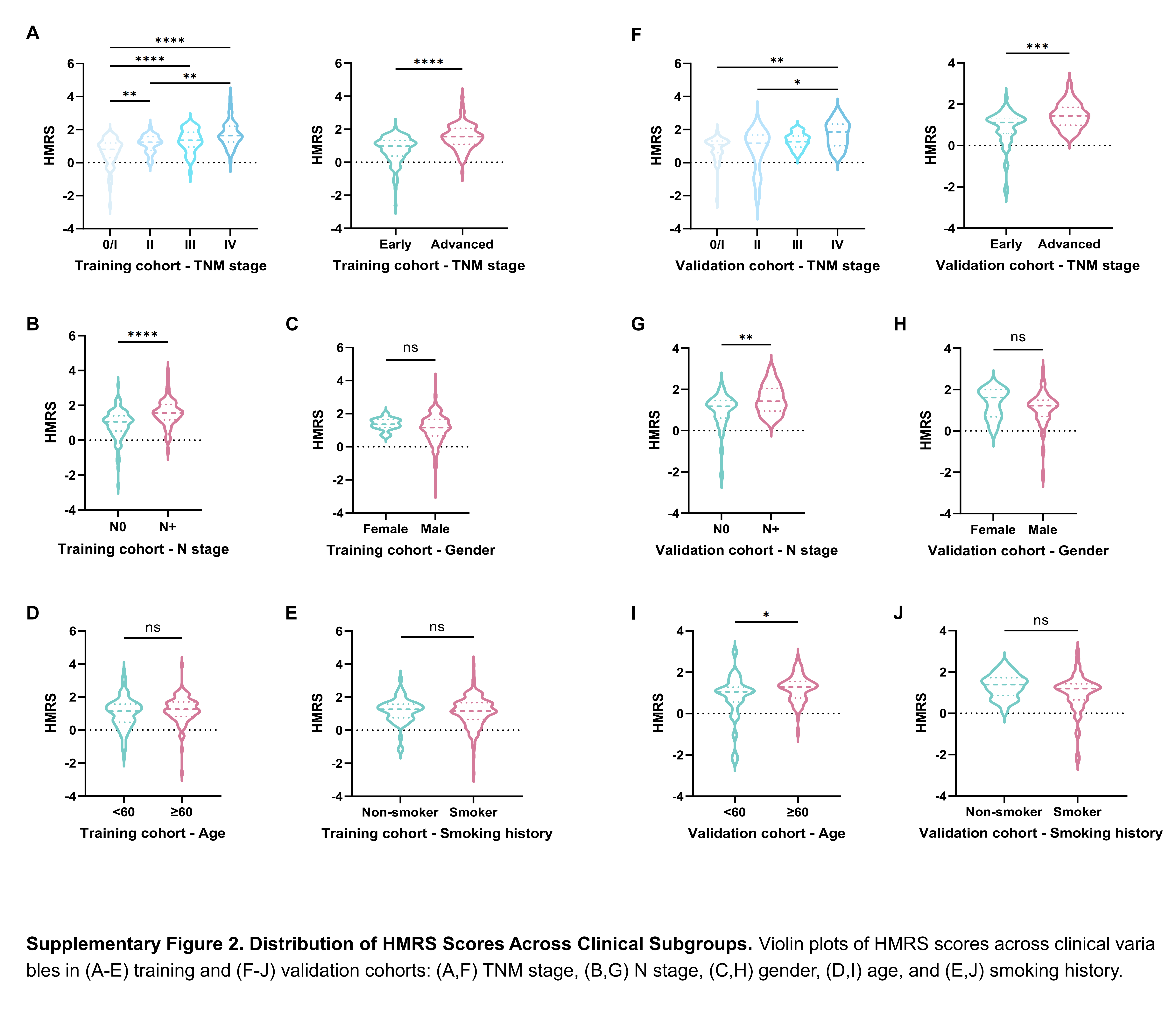

Supplement: Supplementary file 2 [file Image2.tif]

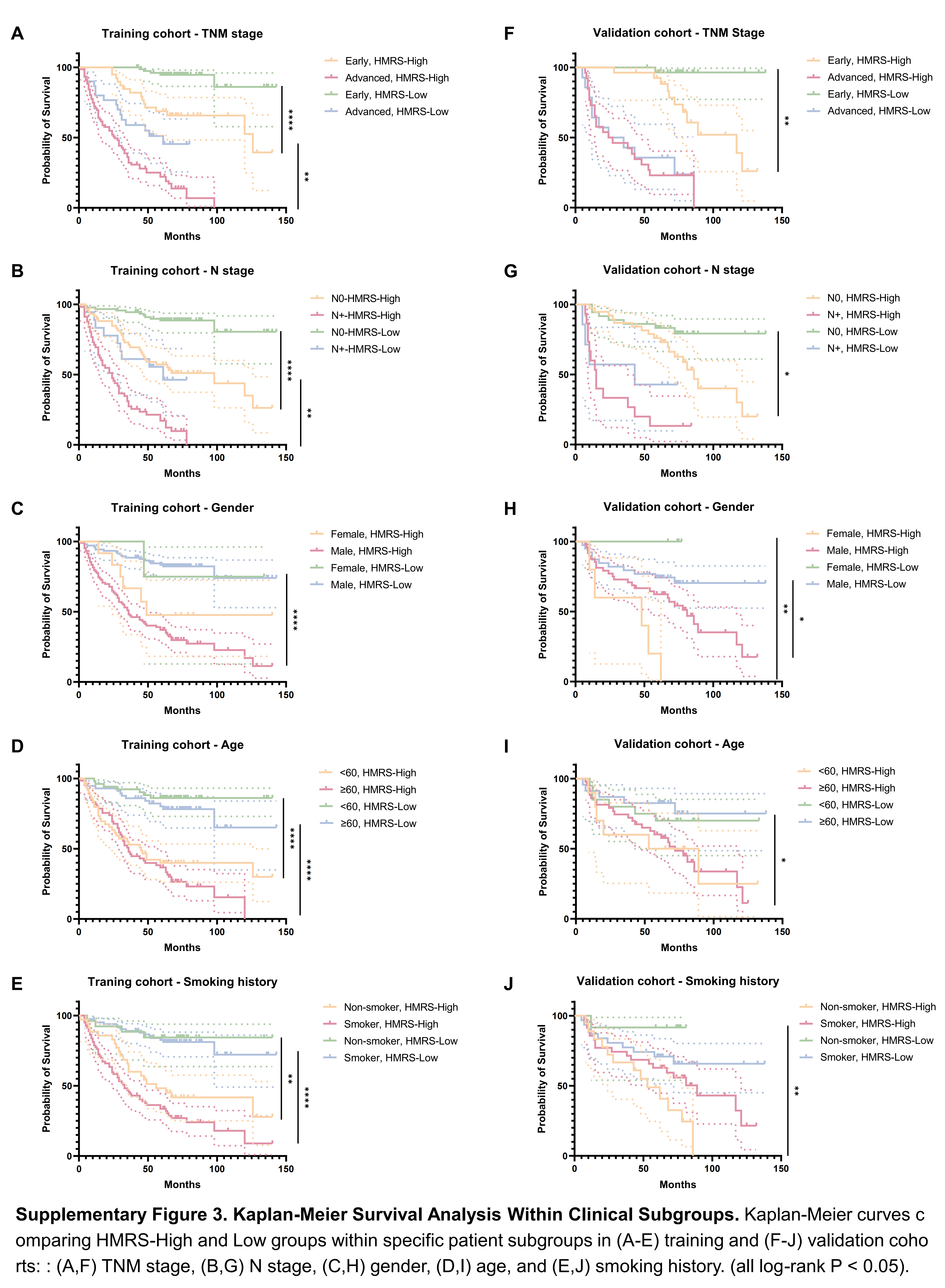

Supplement: Supplementary file 3 [file Image3.tif]

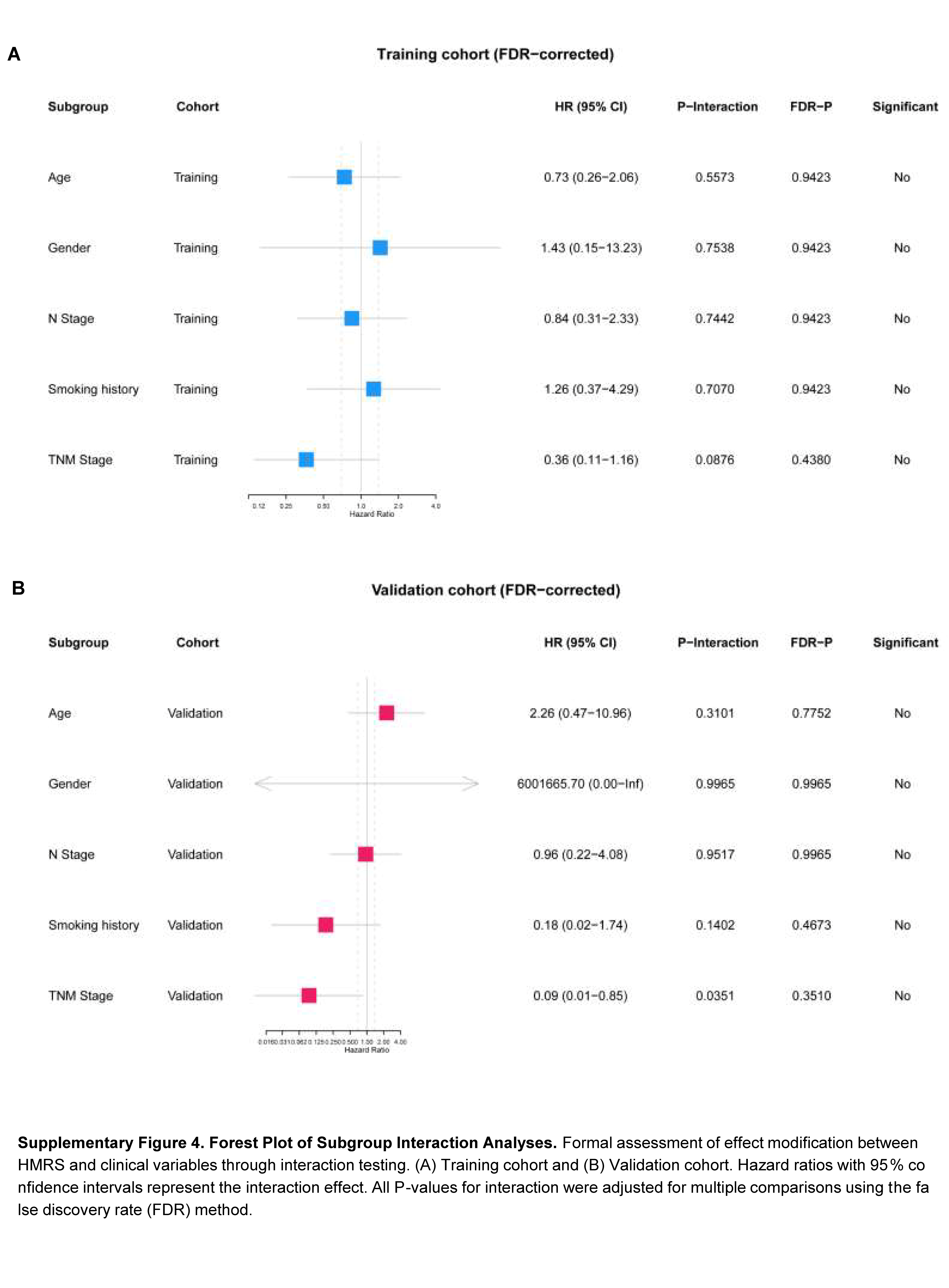

Supplement: Supplementary file 4 [file Image4.tif]

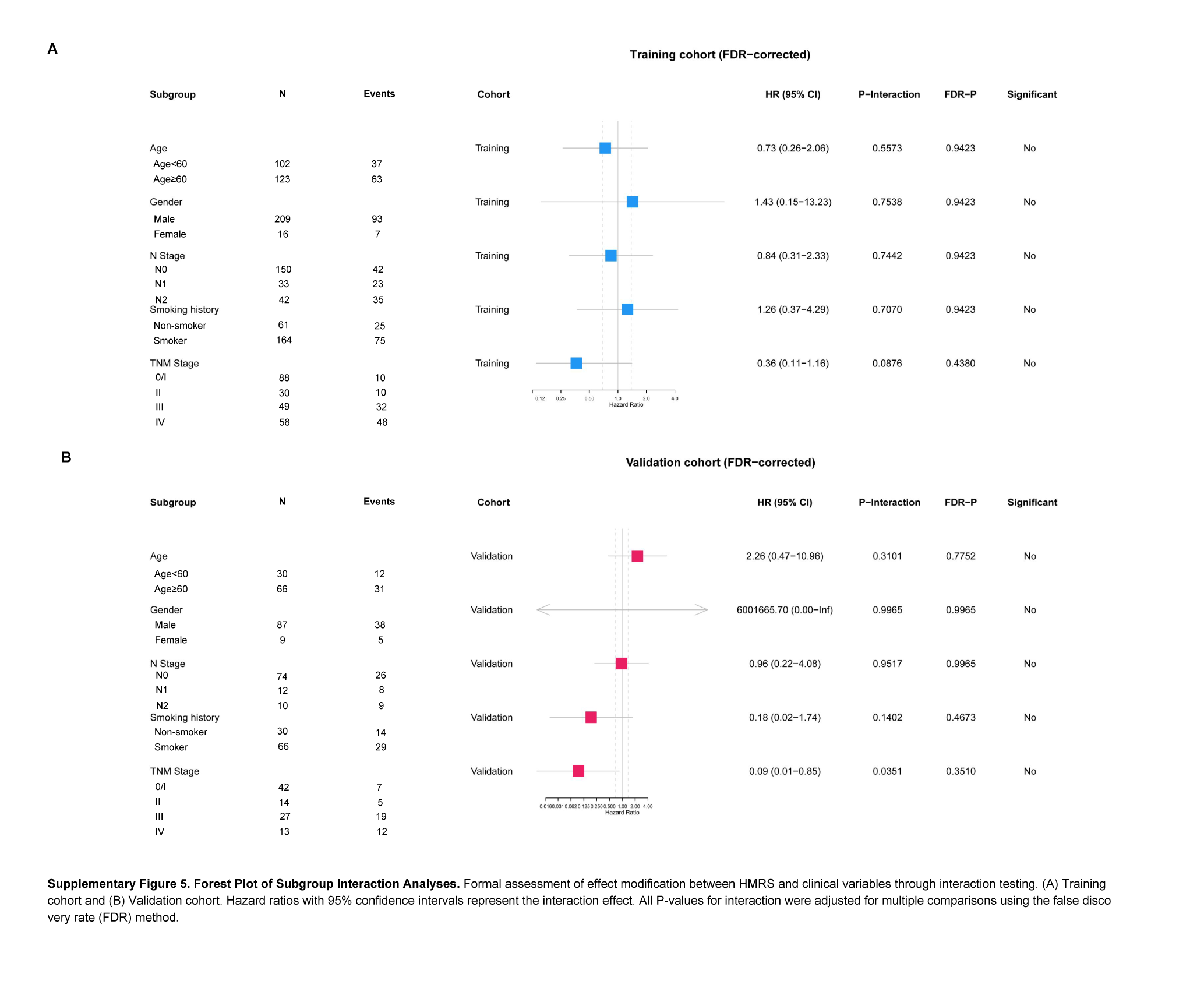

Supplement: Supplementary file 5 [file Image5.tif]

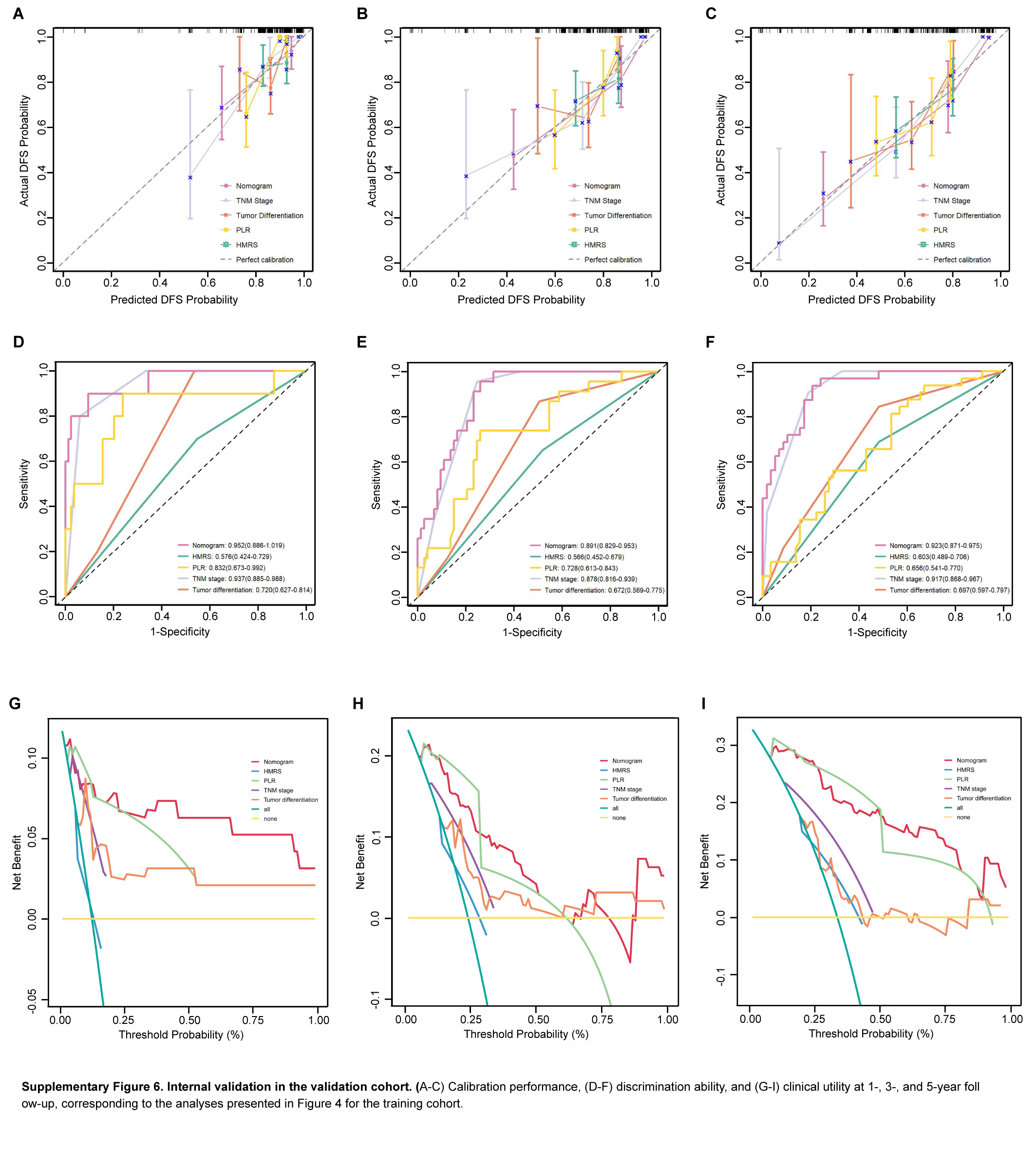

Supplement: Supplementary file 6 [file Image6.tif]
