# Supplementary material for: Development and validation of a novel hepato-metabolic-renal score nomogram for predicting disease-free survival in head and neck squamous cell carcinoma
Source: Front Oncol. 2026 May 21;16:1815660. doi: 10.3389/fonc.2026.1815660 (PMC13233239; doi:10.3389/fonc.2026.1815660)
Supplement: Supplementary file 7 [file Table1.docx]

**Supplementary Table 1.** Sensitivity analysis comparing different HMRS versions in training and validation cohort.

| **Cohort** | **HMRS version** | **Cut-off** | **1-year AUC (95% CI)** | **3-year AUC (95% CI)** | **5-year AUC (95% CI)** |
| --- | --- | --- | --- | --- | --- |
| Training | Original | 1.167 | 0.752 (0.661-0.842) | 0.808 (0.749–0.867) | 0.794 (0.734–0.853) |
| Training | Simplified (6 vars) | -0.158 | 0.759 (0.672–0.845) | 0.765 (0.699–0.831) | 0.747 (0.680–0.815) |
| Training | Clinical (abnormal count) | 2.5 | 0.563 (0.447–0.678) | 0.552 (0.474–0.630) | 0.546 (0.470–0.623) |
| Validation | Original | 1.167* | 0.746 (0.545–0.948) | 0.698 (0.569–0.827) | 0.758 (0.653–0.863) |
| Validation | Simplified (6 vars) | -0.158* | 0.841 (0.730–0.951) | 0.712 (0.577–0.848) | 0.710 (0.590–0.831) |
| Validation | Clinical (abnormal count) | 2.5* | 0.756 (0.650–0.862) | 0.588 (0.459–0.717) | 0.507 (0.388–0.626) |

Note: The original HMRS was derived from LASSO Cox regression using 8 preoperative laboratory parameters (FIB, Cys, Cr, TC, DBIL, ALT, AST, γ-GGT) with z-score standardization. The simplified HMRS excluded variables with coefficients <0.01 (Cr and γ-GGT) to assess their contribution. The clinical HMRS replaced z-score standardization with simple abnormal count (0–8) based on institutional reference ranges to evaluate cross-institutional generalizability. Cut-off values marked with * were derived from the training cohort and applied consistently to the validation cohort.
