# Supplementary material for: Development and validation of a novel hepato-metabolic-renal score nomogram for predicting disease-free survival in head and neck squamous cell carcinoma
Source: Front Oncol. 2026 May 21;16:1815660. doi: 10.3389/fonc.2026.1815660 (PMC13233239; doi:10.3389/fonc.2026.1815660)
Supplement: Supplementary file 8 [file Table2.docx]

**Supplementary Table 2.** Internal validation of the nomogram using bootstrap and cross-validation.

| **Method** | **C-index** | **Calibration slope** | **Optimism** |
| --- | --- | --- | --- |
| bootstrap (1000 resamples) | 0.849 | 0.904 | 0.010 |
| 10-fold cross-validation | 0.858 | 1.117 | - |
